# Risk prediction of two types of potential snail habitats in Anhui Province of China: Model-based approaches

**DOI:** 10.1371/journal.pntd.0008178

**Published:** 2020-04-06

**Authors:** Jun Zhang, Ming Yue, Yi Hu, Robert Bergquist, Chuan Su, Fenghua Gao, Zhi-Guo Cao, Zhijie Zhang

**Affiliations:** 1 Department of Epidemiology and Biostatistics, School of Public Health, Fudan University, Key Laboratory of Public Health Safety of Ministry of Education, School of Public Health, Fudan University, Shanghai, China; 2 Department of Infectious Diseases, The First Affiliated Hospital of Nanjing Medical University, Nanjing, Jiangsu, China; 3 Ingerod, Brastad, Sweden; 4 Center for Global Health, Jiangsu Key Laboratory of Pathogen Biology, Department of Pathogen Biology & Immunology, Nanjing Medical University, Jiangning District, Nanjing, Jiangsu, China; 5 Anhui Institute of Schistosomiasis Control, Hefei, Anhui Province, China; Federal University of Agriculture Abeokuta, NIGERIA

## Abstract

Elimination of the intermediate snail host of *Schistosoma* is the most effective way to control schistosomiasis and the most important first step is to accurately identify the snail habitats. Due to the substantial resources required for traditional, manual snail-searching in the field, and potential risk of miss-classification of potential snail habitats by remote sensing, more convenient and precise methods are urgently needed. Snail data (N = 15,000) from two types of snail habitats (lake/marshland and hilly areas) in Anhui Province, a typical endemic area for schistosomiasis, were collected together with 36 environmental variables covering the whole province. Twelve different models were built and evaluated with indices, such as area under the curve (AUC), Kappa, percent correctly classified (PCC), sensitivity and specificity. We found the presence-absence models performing better than those based on presence-only. However, those derived from machine-learning, especially the random forest (RF) approach were preferable with all indices above 0.90. Distance to nearest river was found to be the most important variable for the lake/marshlands, while the climatic variables were more important for the hilly endemic areas. The predicted high-risk areas for potential snail habitats of the lake/marshland type exist mainly along the Yangtze River, while those of the hilly type are dispersed in the areas south of the Yangtze River. We provide here the first comprehensive risk profile of potential snail habitats based on precise examinations revealing the true distribution and habitat type, thereby improving efficiency and accuracy of snail control including better allocation of limited health resources.

## Author summary

Schistosomiasis is a parasitic disease caused by parasitic worms of the genus *Schistosoma*. In China, the sole intermediate snail host is *Oncomelania hupensis* whose elimination has proved to be the most effective way to interrupt this disease. However, manual snail-searching is labour-intensive, expensive and time-consuming and can lead to inaccurate results. For a better approach, 12 models were employed and compared to characterise the typical snail habitats that vary between the lake/marshlands and the hilly areas. We found that the two types of snail habitats showed notable differences during the modelling process, mainly due to the impact of environmental variables that can form different types of habitats. We further found that habitat characterization contributed to better prediction of areas at risk, and that the precision was high, especially of models based on machine-learning algorithms such as random forest (RF). The highest level of accuracy was achieved by the support vector machine (SVM) approach and artificial neural networks (ANN). Our study provides new insights into accurate prediction of the spatial distribution of potential snail habitats with machine-learning as the preferred approach.

## Introduction

Schistosomiasis is a detrimental parasitic disease caused by parasitic worms of the genus *Schistosoma* [1] and it is prevalent in many parts of the world, including Africa (e.g., Egypt), Asia (e.g., China), and South America (e.g., Brazil) [2,3,4]. The World Health Organization (WHO) regards schistosomiasis as a neglected tropical disease (NTD) and estimates that at least 206.4 million people require preventive treatment for schistosomiasis, out of which only 89 million have been treated (WHO, 2018). In China, all infections are due to *Schistosoma japonicum* with *Oncomelania hupensis* as the sole intermediate snail host which is amphibious rather than aquatic and associated with high moisture microhabitats [5]. In contrast to other schistosome species adapted to humans (*S*. *haematobium*, *S*. *mansoni*, *S*. *intercalatum*, *S*. *guineensis* and *S*. *mekongi*), *S*. *japonicum* not only infects humans but also a wide variety of mammals, particularly domestic animals that act as reservoirs such as the water buffalo [6]. Based on the epidemiological pattern of schistosomiasis and ecological characteristics of the snails, snail habitats can be categorized into three types in China: (i) marshland and lake areas, (ii) mountainous and hilly areas, and (iii) plain areas with waterway networks [7].

After decades of efforts to combat schistosomiasis in China, the number of patients has dropped significantly thanks to mass drug administration (MDA) with praziquantel which controls the morbidity due to schistosomiasis effectively [2]. However, elimination of schistosomiasis requires other methods, in particular control of the transmission of the disease [8]. Surveys conducted in the mid-1950s showed that the snail habitat areas were about 14.3 billion km^2^, which declined to 3.6 billion km^2^ in 2015 [9]. This must decline further but progress in this direction presents complex challenges, which vary depending on the ecology and topography of the endemic areas including lake and marshlands and the hilly or mountainous type. The *O*. *hupensis* snail species is difficult to trace as it is amphibious, generally widely distributed and requires particular environmental conditions, such as high moisture [8]. In the hilly and mountainous areas its distribution is scattered and with often inaccessible habitats making snail control a challenge [10]. In addition, the use of molluscicides is restricted due to environmental protection, resulting in unstable epidemic situations that cannot easily be controlled [11]. Although infected snails are rarely found In recent years [8], the *O*. *hupensis* habitats have not disappeared and some areas even show an upward trend. For example, a snail survey in 2016 covering a total area of 8,140 km^2^ found snails in 2,351 km^2^, including 13 km^2^ of new areas where snails had not been detected before, which meant an increase of 102.16% compared with 2015 [12].

Previous studies have shown when ecological conditions become more favourable, potential snail habitats expand resulting in a general increase in snail numbers, and also an increase in numbers of infected snails [13]. Thus, identification and monitoring of the snail habitats is crucial for effective schistosomiasis control in the long term. This is traditionally carried out through manual snail collection in the field, which has been done on a big scale in China since the 1960s [2,7]. This approach is effective but requires both a large human effort and financial resources. Furthermore, this approach is difficult in less accessible areas, such as hills and marshes. For this reason, remotely sensed environmental data derived from satellite imagery, have been widely used to identify snail habitats, which in combination with actual data on snail distributions as ground truth can be used to predict schistosomiasis risk [14,15]. However, this method can only surmise the existence of snails through the presence of potential habitats and association with particular values of certain environmental variables. Thus the risk of misclassification is high, in particular as the resolution of the satellite optical sensors is generally not sufficient for unequivocal identification of a snail habitat [16]. To improve identification, we therefore attempted a modelling strategy with machine-learning as the approach to achieve superior accuracy on the basis of our original research [17].

## Materials and methods

### Study area

**Fig 1:**
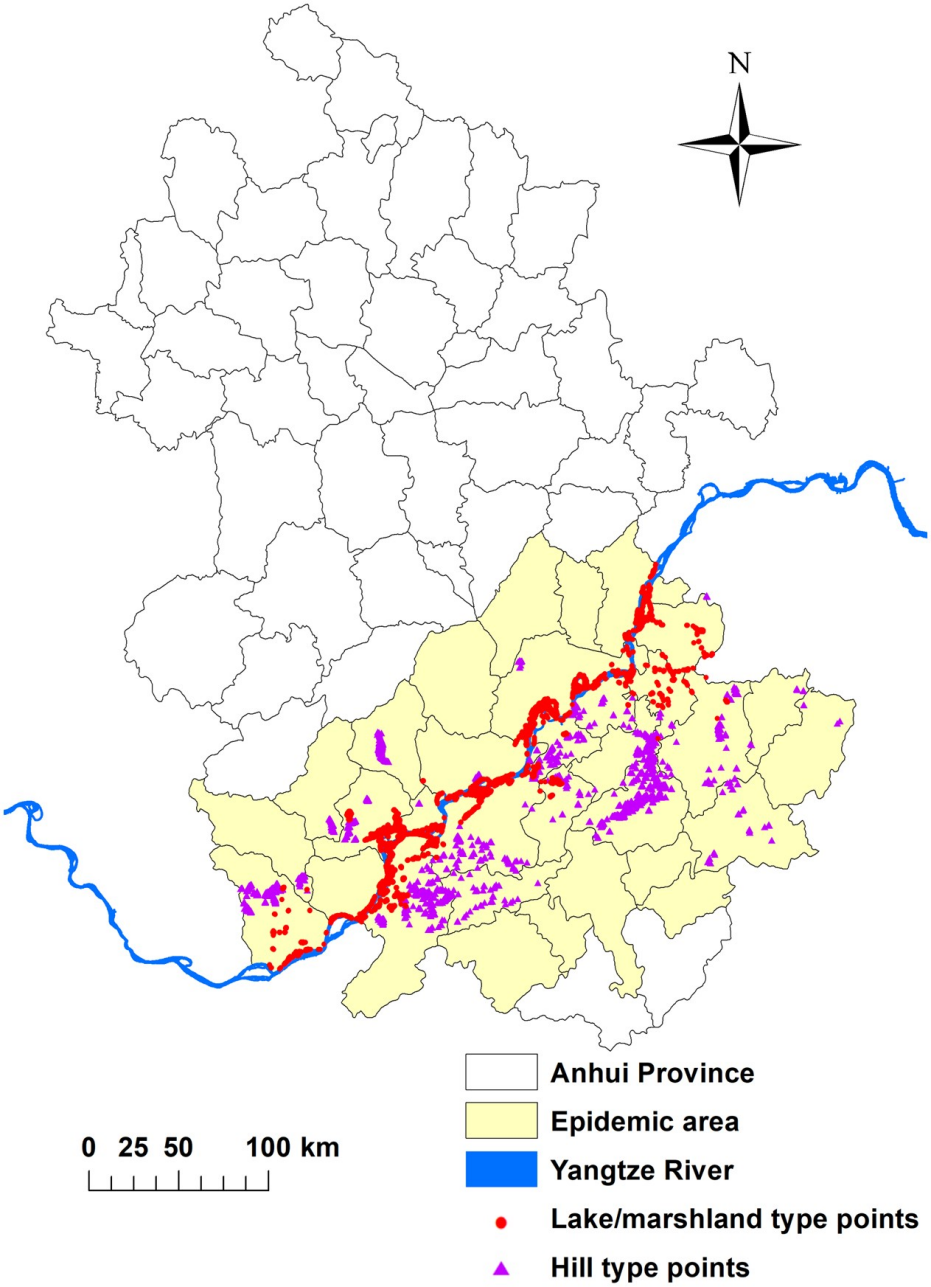
The epidemic areas and sample points in Anhui Province about here. (A) The figure is the epidemic areas of Anhui Province in China with the blue lines through the southern parts of the province representing the Yangtze River. The red and circular purple triangular points represent the sample points in the lake/marshland and hilly/mountainous types of snail habitat, respectively. The map was created using the ArcGIS 10.0 software (ESRI Inc., Redlands, CA, USA).

Anhui is a province located in eastern China between latitudes of 29°41′–34°38′ North, and 114°54′–119°37′ East (Fig 1). Anhui Province has been one of typical epidemic areas of schistosomiasis as a wide range of large lake/marshland and hilly area provide an ideal environment for the growth and reproduction of the intermediate snail host [8]. Anhui Province is one of the most important areas endemic for schistosomiasis and was therefore selected as study area. Past studies have shown that there are four subtypes of oncomelania in different regions of China and the species distributed in Anhui are mainly *Oncomelania hupensis* [10].

### Snail data

The snail distribution data come from the results of a snail survey carried out throughout Anhui Province from March to May, 2016. The snail environment was determined based on the survey from a previous study [18] according to historical data and confirmed in the field by professionals from the local Schistosomiasis Control Station. It was classified into two types of snail habitat, i.e. 1) lake and marshlands; and 2) hilly and mountainous areas (Fig 1) [19]. Past studies have shown that there are four subtypes of *Oncomelania* in different regions of China and the species distributed in Anhui is mainly *O*. *hupensis* [10].

The exact points where the snails were found or historically reported were located by a handheld global positioning system (GPS) instrument (Garmin GPSMAP 64s) [20]. For areas where no snail habitats were found, a random sampling method was applied with a buffer area of 100m to exclude them from the snail habitat areas of interest [21].

### Environmental data

Thirty-six environmental variables were included in our study (see Table 1 for variable summaries). Most of the environmental variables were remotely sensed from Earth-orbiting satellite sensors. We calculated the Normalized Difference Vegetation Index (NDVI) and the land surface temperature (LST) [21] based on the satellite images of our study area. The elevation data (DEM) was obtained from the Global Land Information System (GLIS) of the United States Geological Survey (USGS) and we extracted aspect (Asp) and slope accordingly [22]. The distance to nearest water body (Water), which includes rivers and lakes in the whole study area, was calculated from water body data that were downloaded from Conservation Science Data Sets of World Wildlife Foundation. The climatic variables were Bio1~Bio19 obtained from WorldClim (see S1 Table for details) [23,24]. The other climatic variables and soil data, geomorphic type (Geo), land use type (Lucc), ecosystem type (Eco) and vegetation type (Veg) all came from the Data Center for Resources and Environmental Sciences of the Chinese Academy of Sciences.

**Table 1:** Summary of environment variables used in study before screening.

Data description	Label	Variable type
Normalized Difference Vegetation Index	NDVI	Continuous
Land surface temperature	LST	Continuous
Elevation	DEM	Continuous
Aspect	Asp	Continuous
Slope	Slope	Continuous
Distance to nearest water body	Water	Continuous
WorldClim	Bio1~Bio19	Continuous
Accumulated temperature beyond 0°C	Aat0	Continuous
Accumulated temperature beyond 10°C	Aat10	Continuous
Moisture index	Im	Continuous
Annual average precipitation	Pa	Continuous
Annual average temperature	Tadem	Continuous
Soil type	Soil	Categorical
Soil texture	Clay, Sand, Silt	Continuous
Geomorphic type	Geo	Categorical
Land use type	Lucc	Categorical
Ecosystem type	Eco	Categorical
Vegetation type	Veg	Categorical

### Data preprocessing

After representing the study area as a map with a 100×100 m matrix grid, the 10,000 m^2^ wide cells where snail habitats had been found were marked as ‘1’ with the centre of the cell as their location, alternatively with ‘0’ if no snail habitat had been recorded. For the modelling, the re-located locations (cell centres) were used, not the actual location, since the map of the whole area was needed for the prediction [18]. According to the type of habitat environment, we divided the habitat presence points into two groups: 1) lake/marshlands and 2) hilly and mountainous areas. To ensure that the environmental raster data had the same geographical scope and same scale as the study area, we used the polygon of Anhui Province as the mask for all environmental data and then converted them into the form of raster image with the same scale.

To control the potential multi-collinearity among the environmental variables, correlation analysis was conducted for all climate raster images to gain the correlation coefficients for the matrices. One of the variables was excluded from every pair of variables with a correlation coefficient greater than 0.7 [25]. Which variables to be excluded depended on the results of the *t* test for the pair of variables with respect to the two groups (presence and absence), preserving the variable with the lower *P*-value (i.e. the one with the most statistically significant correlation with *Oncomelania* environment). We conducted screening of all variables for the two types of habitat, i.e. lake/marshlands and hilly/mountainous areas.

### Modelling and evaluation

Twelve models were produced: two based on sections (Bioclim and Domain), three on traditional statistical methods, such as generalized linear model (GLM), multivariate adaptive regression spline (MARS) and flexible discriminant analysis, (FDA), and seven on machine-learning algorithms, such as maximum entropy, Maxent), Genetic Algorithm for Rule-set Production (GARP), generalized boosted models (GBM), random forest (RF), classification tree analysis (CTA), artificial neural network (ANN) and support vector machine (SVM).

Four of the models (Bioclim, Domain, Maxent and GARP) are presence only models and they only need the set of presence records. Bioclim and Domain from DIVA-GIS software, version 7.1.7 (http://www.diva-gis.org/) were developed by constraining the range of environmental factors [26]. While Maxent and GARP are machine-learning programs, the former conducted by the stand-alone Maxent software, version 3.4.1 [27], and the latter based on biological evolution theory, which also has its stand-alone software, i.e. Desktop GARP, version 1.1.6 [28]. The other eight models, were presence/absence models which need both presence and absence records: ANN and SVM were built in R, version 3.5.1 by the *nnet* package and the *e1071* package, respectively, while the other six presence/absence models were carried out using the *Biomod2* package for R [29] (more details are shown in appendix S1 Text).

The whole dataset was split randomly into two parts, 75% for model development and 25% for model evaluation. The 12 models were compared with the five evaluation indices applied for model testing, including sensitivity, specificity, percent correctly classified (PCC), Kappa and area under the (receiver operating characteristic) curve (AUC) [30]. They were performed with the *Presence/Absence* packages with a 0.5 threshold, where >0.5 represented the potentially positive areas (snails present) and <0.5 the potentially negative areas (snails absent) [31]. Besides, the importance of variables would be evaluated and compared in the best models, which is RF variables importance algorithm and its principle is to shuffle a single variable of the given data. The higher the value of the importance of variable (IV), the more influence the variable would have on the model.

## Results

In total, 45,000 randomly sampled points delineating presence/absence of snails were included comprising. 5,000 presence points and 10,000 absence points for the lake/marshland area, and 10,000 presence points and 20,000 absence points for the hilly/mountainous area.

Importantly, snails in two types of habitats showed preferences with respect to the climate variables, as seen in the matrix of correlation coefficients (S3 and S4 Figs). For the lake/marshlands, Bio3, Bio6, Bio8, Bio9 and the accumulated temperature beyond 10°C (Aat10) were statistically significant, while for the hilly/mountainous areas, the moisture index (Im), and Bio8, Bio9, Bio12, Bio15 and the accumulated temperature beyond 0°C (Aat0).

**Fig 2:**
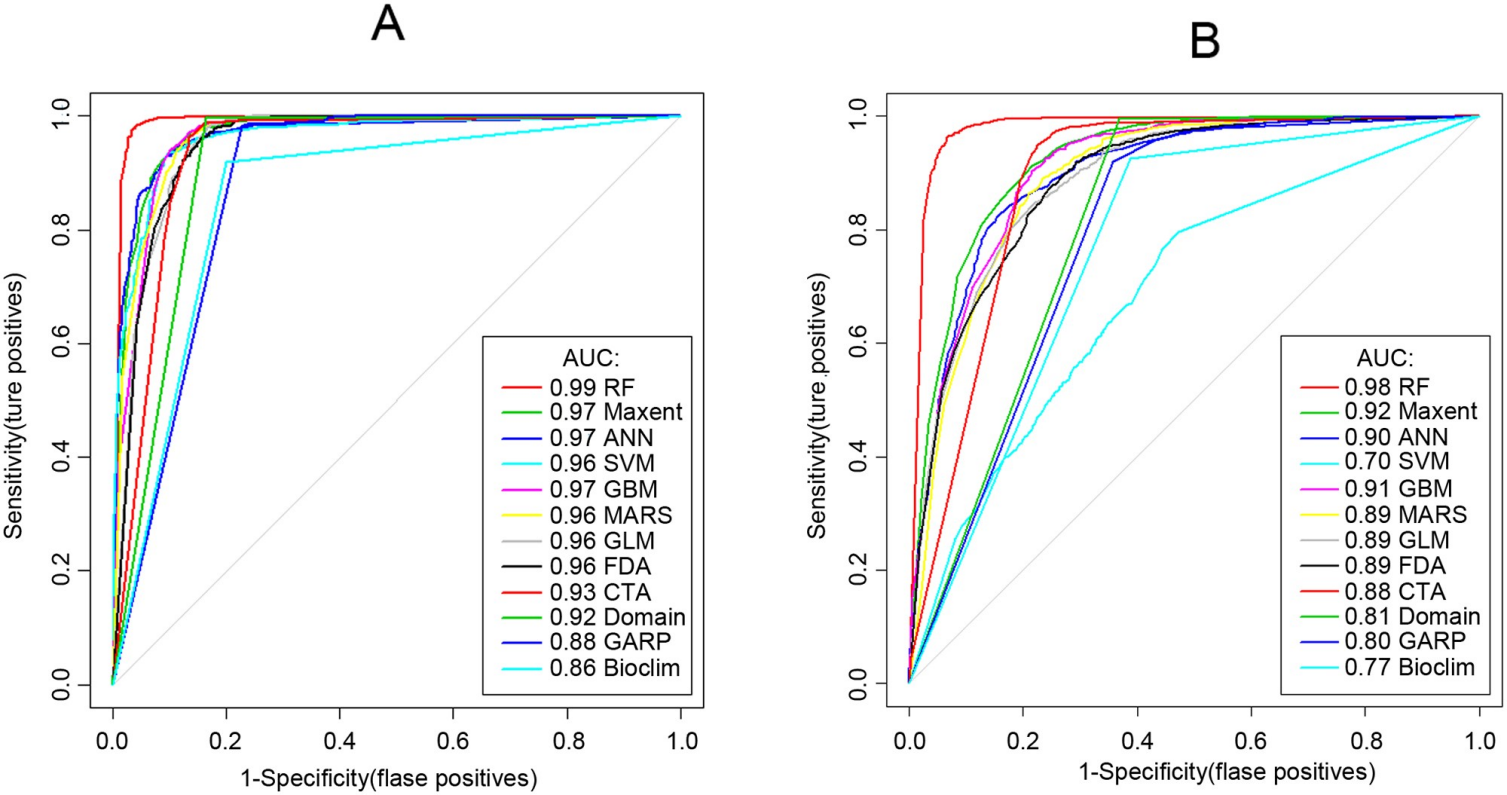
ROC curves of predicted results of the 12 models for the two types of snail habitat. (A) ROC curve for snail habitats in the lake/marshlands. (B) ROC curve for snail habitats in the hilly/mountainous areas.

Judging from the results of the various predictive indicators (Table 2) and the ROC curve (Fig 2), there was no difference in ranking of the model effects between the two types of geographical area. However, the predictions with regard to the lakes and marshlands area were slightly better than those with regard to the hilly and mountainous areas. Generally, the presence/absence models outperformed the presence only models, especially the models based on machine-learning algorithms such as RF, SVM and ANN. In the light of AUC, Kappa and PCC, the prediction results of presence/absence models were better than those based on the presence-only models. Although the AUC of Maxent was high and had the best specificity, its sensitivity was the lowest. With respect to RF, all evaluating indicators except the sensitivity one were the best of 12 models, giving an AUC of this model for the two types of snail habitat of 0.988 and 0.985, respectively, followed by SVM, ANN and Maxent, while Domain, Bioclim and GARP showed higher sensitivity but much lower specificity. Hence, RF was selected as the prediction model of choice.

**Table 2:** Evaluation of 12 models based on five different statistical indexes.

		AUC		Kappa		PCC		sensitivity		specificity	
Model	PO*/PA**	lake/marshland	hilly	lake/marshland	hilly	lake/marshland	hilly	lake/marshland	hilly	lake/marshland	hilly
RF	PA	0.993	0.985	0.928	0.883	0.964	0.942	0.991	0.974	0.937	0.908
ANN	PA	0.970	0.900	0.809	0.618	0.905	0.809	0.857	0.712	0.952	0.887
SVM	PA	0.962	0.925	0.762	0.638	0.881	0.819	0.826	0.731	0.936	0.907
Maxent	PO	0.969	0.922	0.656	0.638	0.828	0.820	0.680	0.724	0.974	0.914
GBM	PA	0.965	0.910	0.802	0.666	0.900	0.833	0.986	0.951	0.816	0.715
MARS	PA	0.964	0.895	0.810	0.634	0.905	0.817	0.983	0.948	0.828	0.686
GLM	PA	0.955	0.894	0.800	0.602	0.900	0.801	0.984	0.941	0.817	0.661
FDA	PA	0.955	0.891	0.799	0.623	0.900	0.812	0.944	0.897	0.856	0.727
CTA	PA	0.935	0.885	0.822	0.716	0.911	0.858	0.987	0.973	0.836	0.744
Domain	PO	0.916	0.813	0.832	0.626	0.916	0.813	0.997	0.995	0.835	0.631
GARP	PO	0.882	0.796	0.625	0.349	0.812	0.675	0.991	0.988	0.635	0.362
Bioclim	PO	0.860	0.768	0.719	0.536	0.860	0.768	0.920	0.924	0.800	0.613

*Presence only;

**Presence and absence

Table 3 shows the importance of variables (IV) in the RF model, i.e. the one found to be the best. For the lake/marshlands, the most important variable for predicting the potential snail habitats was the distance to the nearest river (Water) (IV = 0.305), followed by some climatic variables, such as mean temperature of the driest quarter (Bio9), accumulated temperature beyond 10°C (Aat10) and isothermality (Bio3). However, the most important variables associated with the potential of snail habitats in the hilly areas were two climatic variables (i.e. mean temperature of driest quarter (Bio9) (IV = 0.362) and annual precipitation (Bio12) (IV = 0.335), followed by elevation (DEM) and the vegetation type (Veg).

**Table 3:** The importance of variables (IV).

Lake/marshland type		Hilly type	
Variable name	Importance of variables (IV)	Variable name	Importance of variables (IV)
Water	0.305	Bio9	0.362
Bio9	0.268	Bio12	0.335
Aat10	0.190	DEM	0.121
Bio3	0.035	Veg	0.118
DEM	0.032	Bio8	0.072
Im	0.015	Water	0.047
Bio6	0.010	Aat0	0.034
LST	0.008	Bio15	0.017
Geo	0.007	Soil	0.013
Bio8	0.004	NDVI	0.009
Clay	0.004	LST	0.007
NDVI	0.004	Slope	0.007
Lucc	0.003	Clay	0.006
Veg	0.002	Geo	0.005
Asp	0.001	Sand	0.002
Eco	0.001	Asp	0.001
Silt	0.001	Eco	0.001
Slope	0.001	Lucc	0.001
Soil	0.001	Silt	0.001
Sand	0.000		

**Fig 3:**
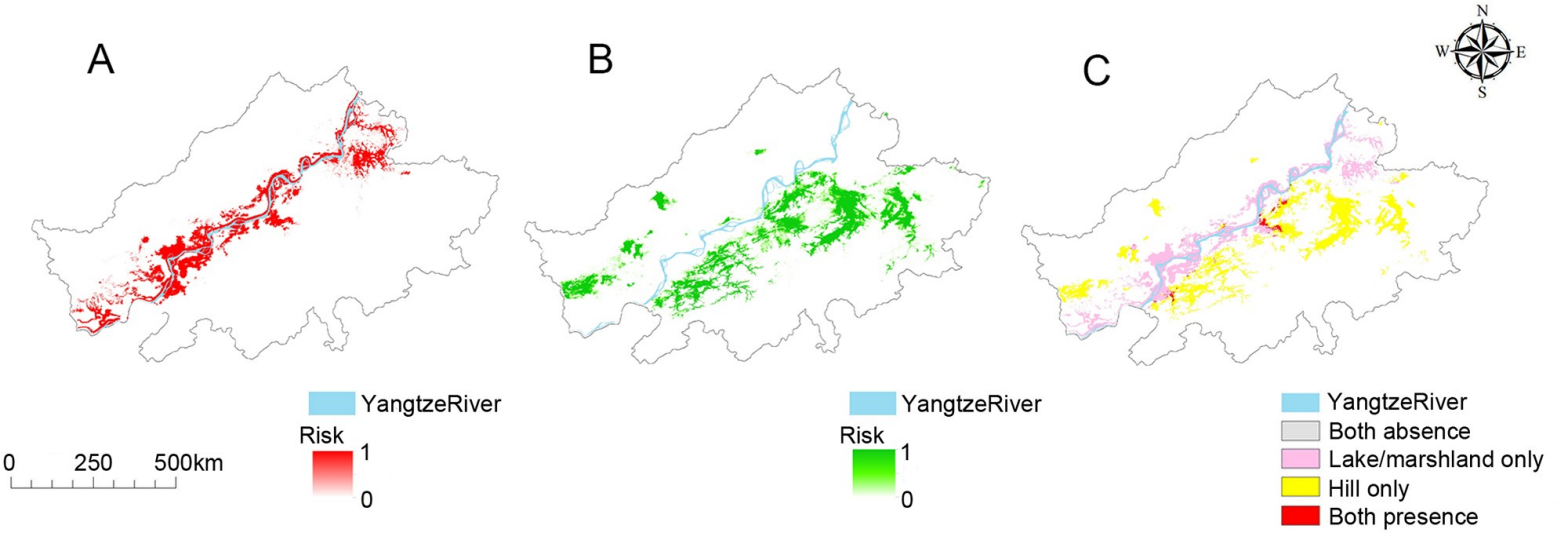
Predicted risk map of potential snail habitats for Anhui Province according to the RF model. (A) Risk map of potential snail habitats for the lake/marshland type. The shifting shades of the colour red from light to dark represent the risk of snail presence changing from low to high. (B) Risk map of potential snail habitats for the hilly/mountainous type. The shifting shades of the colour green from light to dark represent the risk of snail presence changing from low to high. (C) Combined risk map with pink areas representing the lake/marshland type only, yellow areas the hilly/mountainous type only, and the red areas the overlapping regions.

The final risk maps for the potential snail habitats are depicted in Fig 3. The potentially positive areas of snail habitats for the type of lake/marshland area are mainly along the Yangtze River (Fig 3A), while those of the hilly/mountainous areas were more dispersed and mostly distributed in the areas south of the Yangtze River (Fig 3B). The predicted area of snail habitats for these two types of area was 3,712.4 km^2^ and 3,693.1 km^2^ respectively, but the latter was more dispersed. There was also a 122.2 km^2^ overlap of the two high risk areas in the two types of habitat when the prediction results were viewed together on the integrated risk map (Fig 3C).

## Discussion

So far, prediction of potential snail habitats has been widely based on the method of Maxent alone [31,32]. Recent studies have used remotely sensed data and several environmental data to derive habitat suitability for *Bulinus* and therefore *haematobium* risk within African settings [33, 34]. Today, China is moving toward control and elimination of *S*. *japonicum* and precision mapping could play a key role in the more targeted interventions needed in the future [35]. However, it should be considered that it is more difficult to map amphibious snails than the aquatic species, that play the role of intermediate hosts in Africa and Latin America, as the former can potentially be dispersed over a much wider area, which means that highly accurate modelling techniques are of crucial importance for *S*. *japonicum* transmission. In our study, we predicted the potential distribution of *O*. *hupensis* in Anhui Province for two types of snail habitats based on 12 models with a thorough comparison. We found that presence/absence models are better than presence only models and that machine-learning approaches are generally better than other methods, with RF performing best among all the models investigated. Our prediction maps of potential snail habitats should be valuable for directing local staff to conduct precise snail investigations, which would increase the efficiency and accuracy of monitoring snail habitats, particularly when the climate varies from the traditional.

Seen as a whole, the 12 models (S3 and S4 Figs) share certain similar outcomes, such as indicating a concentration of high-risk areas in the southern part of Anhui Province. The high-risk regions for the lake/marshland type were found to be close to the Yangtze River, while those in hilly/mountainous areas were widely scattered, which is consistent with the known situation for *O*. *hupensis* in Anhui Province [20]. However, there were differences among the models. The high-risk areas indicated by some of them, e.g., GARP, were large and dispersed, while Maxent and Domain showed smaller high-risk areas but large median-risk areas (risk around 0.5) which may lead to misclassifications. Compared with the presence only models, the high-risk areas of presence/absence models were more concentrated and there was almost no median-risk area indicated, which may be due to the fact that information of absence data was used and hence improved the accuracy of the model predictions. Although, the Maxent model has been historically used to model potential snail habitats, its lower sensitivity suggests that it should be replaced by better models such as RF. Besides, we found that the ROC for lake/marshland specificity is less variable between models than for hilly mountainous areas, which may have resulted from greater sample sizes from the lake/marshland areas. Sample size is very likely a factor worthy of further investigation with regard to prediction accuracy.

Significant differences were found when two types of *O*. *hupensis* habitats were modelled separately. The difference between the two types of environments was found to be directly reflected by the outcome of variable screening. The most important factor for the lake/marshland areas was found to be distance to the nearest river, but in case of the hilly/mountainous areas, climatic variables were more significant. This is reasonable since the former land is characterized by the ecological features termed “land in winter—water in summer” and “no snails if no vegetation”, while the latter is more related to the local environment’s soil humidity which is closely related to climatic factors [36]. Besides, we found that the predictions performed better for lake/marsh areas than mountainous areas, which might be because the snails in the former are more sensitive to the environment. The overriding significance of the work presented here is that the environmental factors generate different predictions with regard to snail distributions depending on the type of area investigated, which means that predicted results with regard to potential snail habitats must be adapted to the specific area under scrutiny. Our results presented here suggest that the same snail species may produce different outcomes in different living environments, but further research is needed to confirm this finding.

Although a number of variables were used in our study, most of them abiotic, human factors and related issues, such as economy, population, urbanization and environment reconstruction should not be ignored [37,38,39]. For instance, some water conservancy projects such as the South-to-North Water Diversion (SNWD) project are believed to influence the distribution of *O*. *hupensis* [40]. Besides, traditional niche models, including Maxent and GARP, could not meet our increasing need for prediction, because the relation between snail and environment required larger data volumes, which would increase model complexity. Therefore, more flexible prediction models need to be considered and developed in the future, especially with respect to models based on machine-learning algorithms which are currently the most promising direction for the identification of potential snail habitats. Our study provides new insights into how to achieve accurate prediction of the spatial distribution of potential snail habitats with machine-learning as the preferred approach, and it provides also guidance regarding public health approaches for the control of schistosomiasis.

## Supporting information

S1 Fig Correlation matrix of variables for lake/ marshland type areas. (TIF)

S2 Fig Correlation matrix of variables for hilly type areas. (TIF)

S3 Fig Risk maps of 12 models for lake/ marshland type areas. Risk maps of all 12 models of potential snail habitats for the lake/marshland type. The closer the colour of area is to red, the higher the risk for an area of being a snail habitat. Similarly, the closer the colour is to green, the higher the risk of the area being a snail habitat.
(TIF)

S4 Fig Risk maps of 12 models for hilly type areas. Risk maps of all 12 models of potential snail habitats for the hilly type. The closer the colour of area is to red, the higher the risk of it representing snail habitats. Similarly, the closer the colour is to green, the higher the risk of the area being a snail habitat.
(TIF)

S1 Table Explanation of the WorldClim climatic variables. (DOCX)

S1 Text Detailed introduction of models used in this study. (DOCX)

S2 Text The detail of the investigation of Oncomelania hupensis snails in Anhui Province. (DOCX)
