# Supplementary figures and images for: Risk prediction of two types of potential snail habitats in Anhui Province of China: Model-based approaches
Source: PLoS Negl Trop Dis. 2020 Apr 6;14(4):e0008178. doi: 10.1371/journal.pntd.0008178 (PMC7162538; doi:10.1371/journal.pntd.0008178)

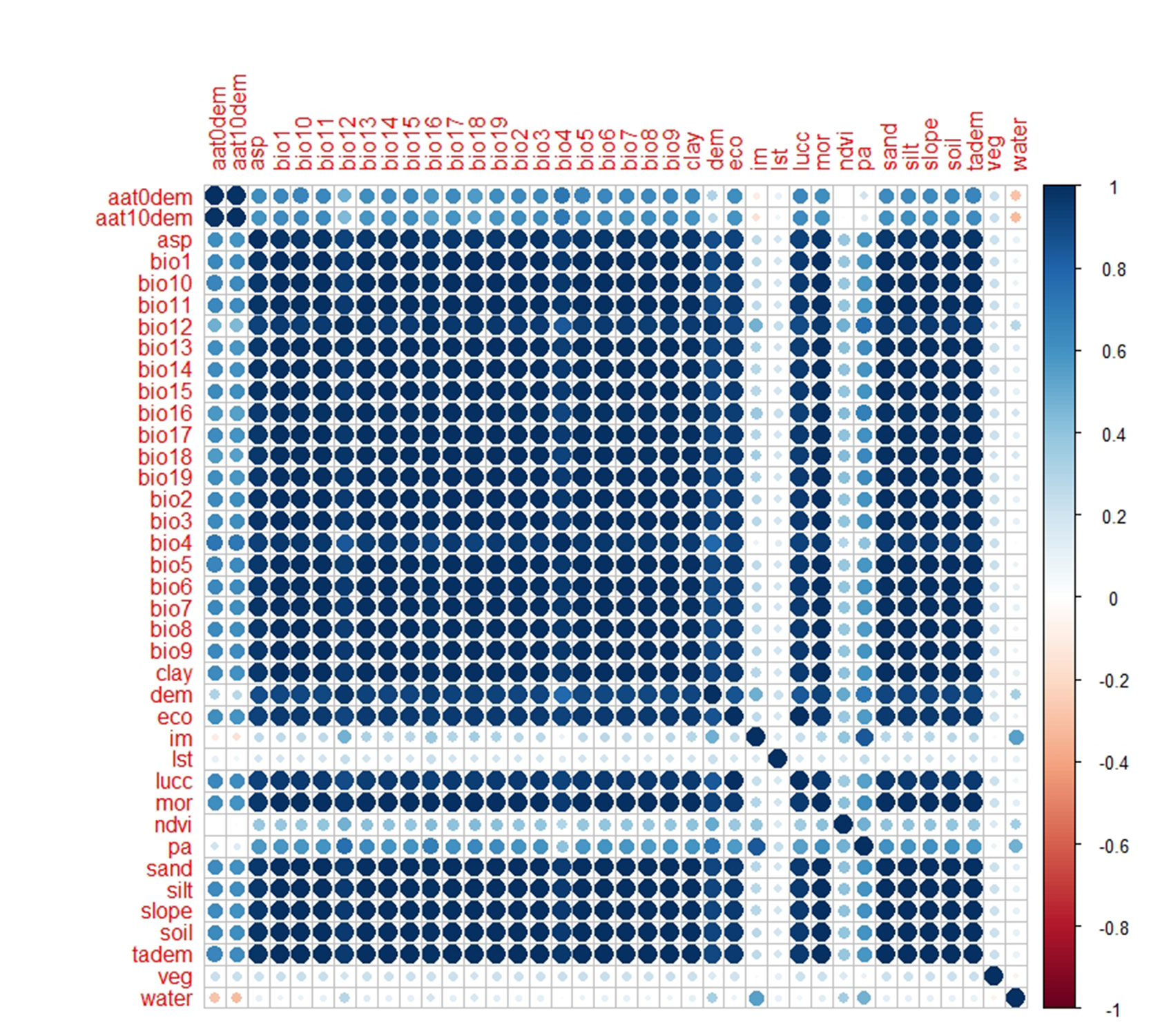

Supplement: S1 Fig — (TIF) [file pntd.0008178.s001.tif]

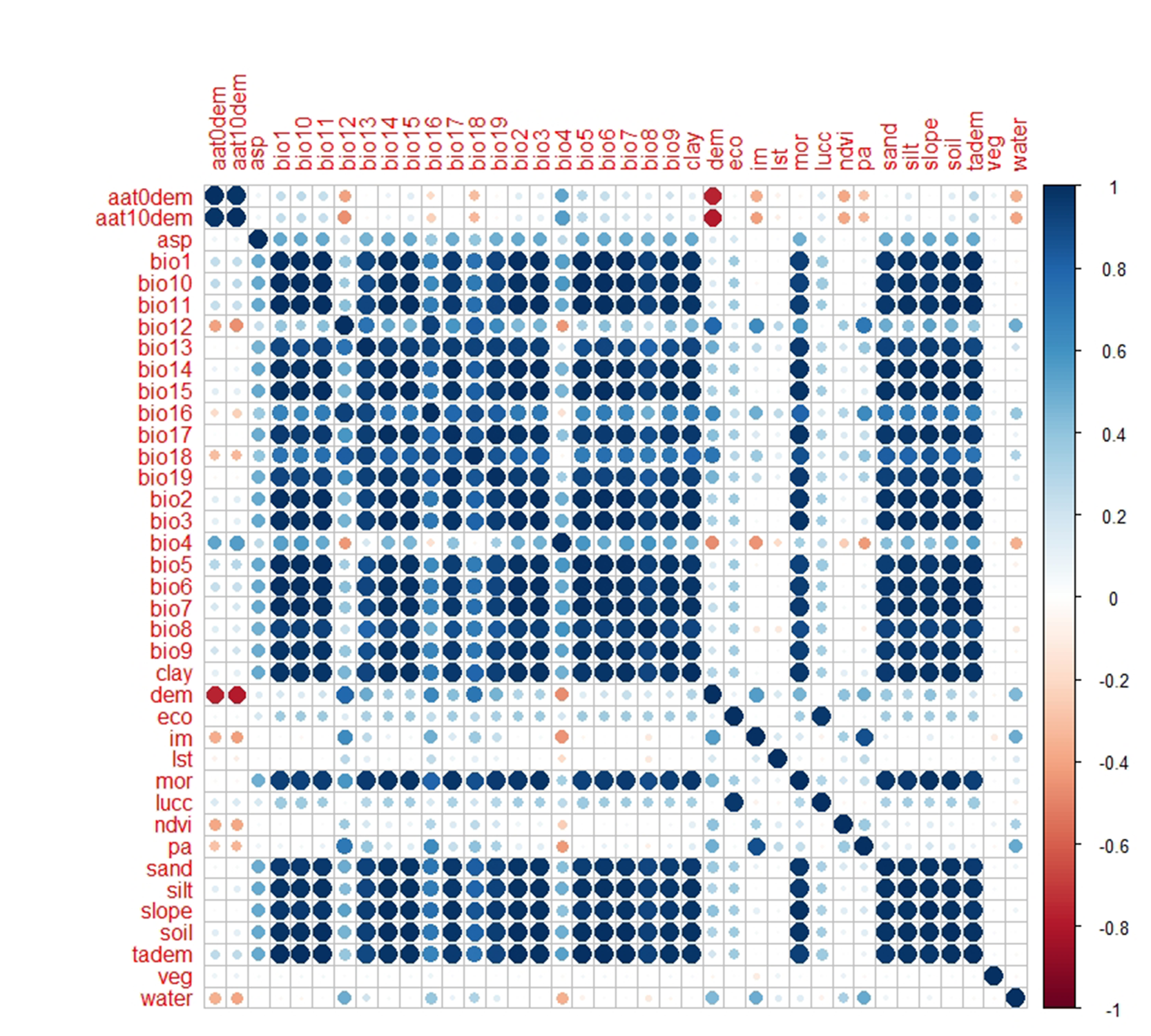

Supplement: S2 Fig — (TIF) [file pntd.0008178.s002.tif]

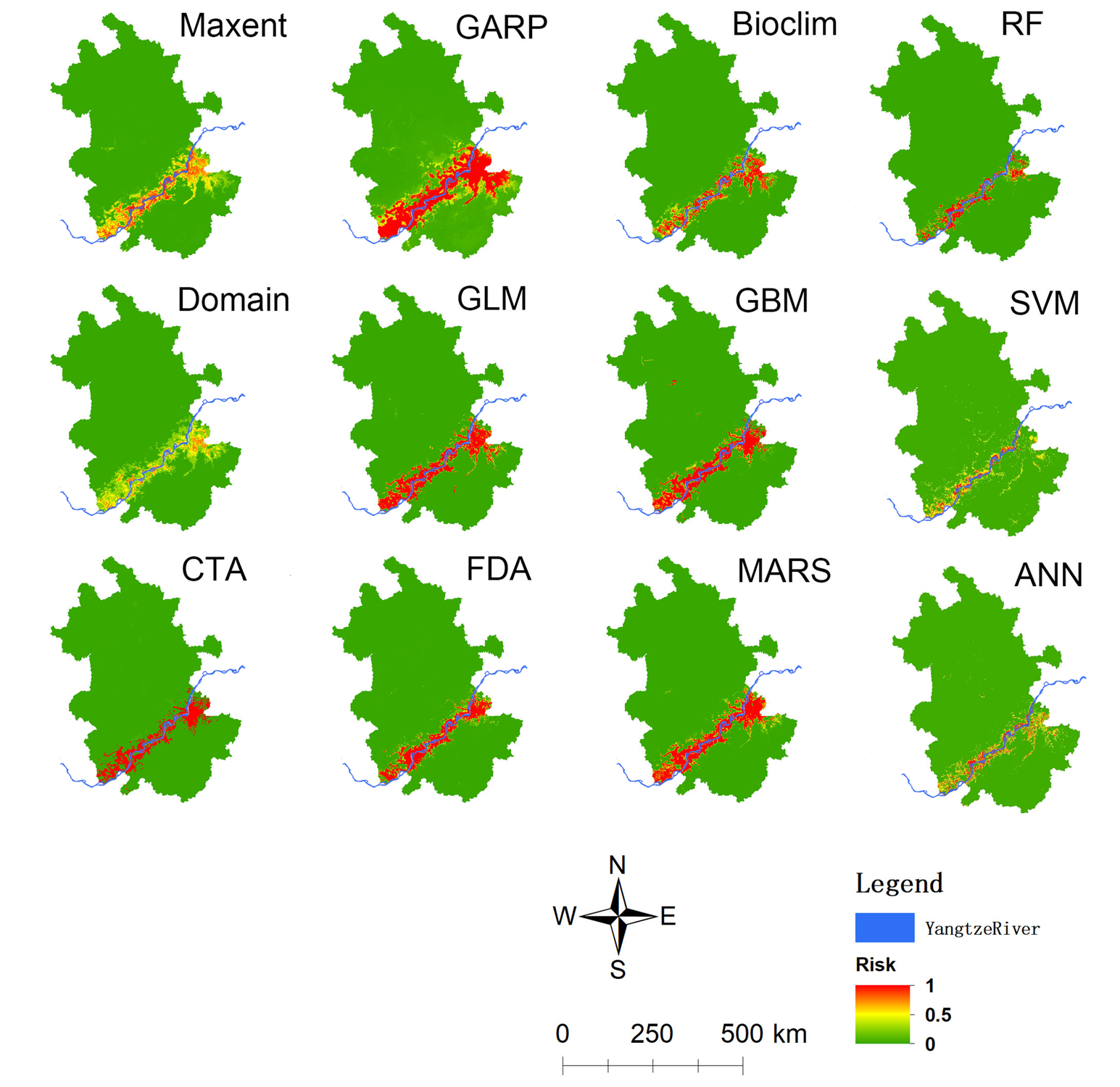

Supplement: S3 Fig — Risk maps of all 12 models of potential snail habitats for the lake/marshland type. The closer the colour of area is to red, the higher the risk for an area of being a snail habitat. Similarly, the closer the colour is to green, the higher the risk of the area being a snail habitat. (TIF) [file pntd.0008178.s003.tif]

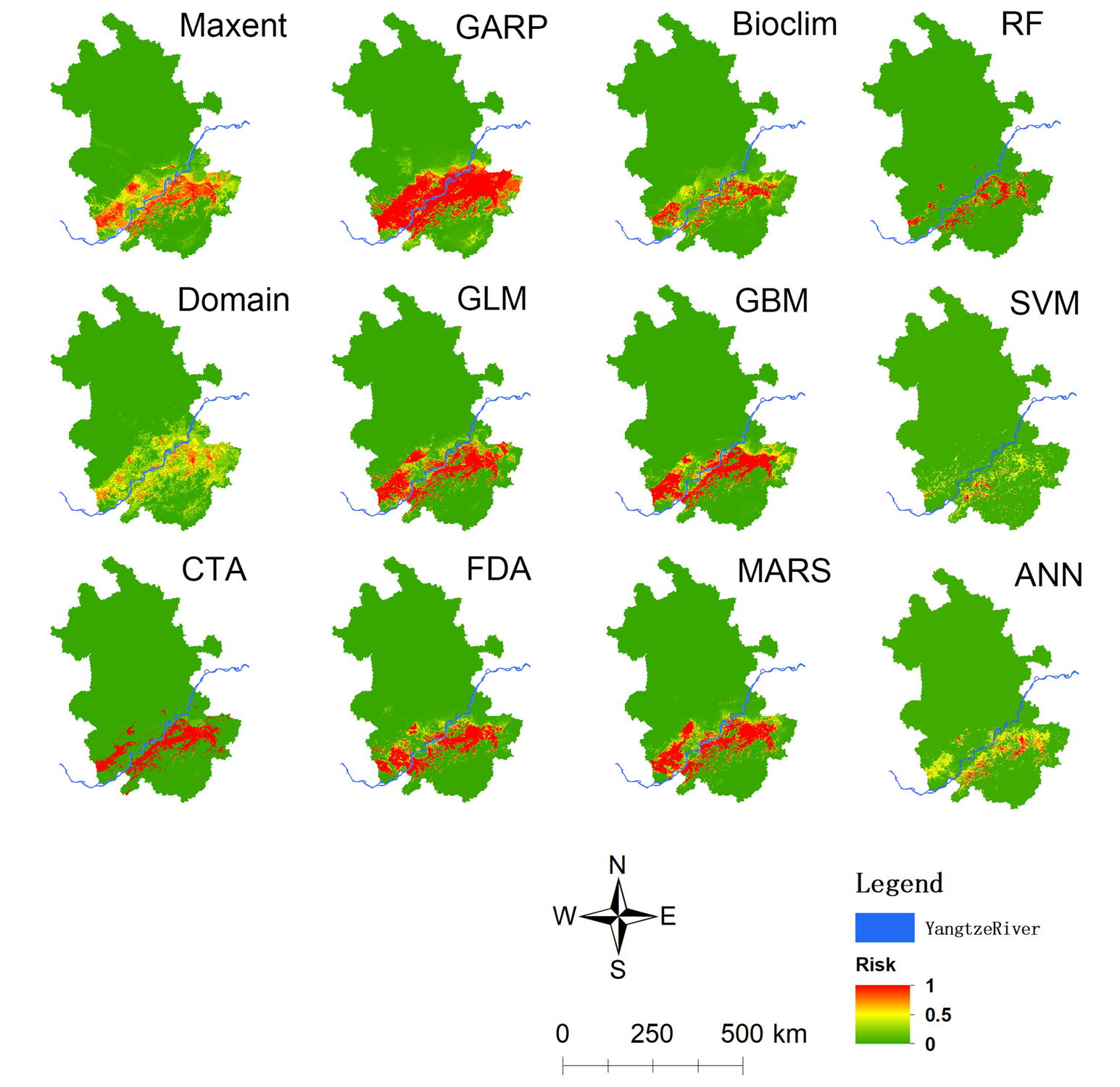

Supplement: S4 Fig — Risk maps of all 12 models of potential snail habitats for the hilly type. The closer the colour of area is to red, the higher the risk of it representing snail habitats. Similarly, the closer the colour is to green, the higher the risk of the area being a snail habitat. (TIF) [file pntd.0008178.s004.tif]
