# Supplementary material for: Risk prediction of two types of potential snail habitats in Anhui Province of China: Model-based approaches
Source: PLoS Negl Trop Dis. 2020 Apr 6;14(4):e0008178. doi: 10.1371/journal.pntd.0008178 (PMC7162538; doi:10.1371/journal.pntd.0008178)
Supplement: S1 Table — (DOCX) [file pntd.0008178.s005.docx]

S1 Table. Meanings of the Climatic Variables from WorldClim.

| Various label | Data description |
| --- | --- |
| Bio1 | annual mean temperature |
| Bio2 | mean diurnal range |
| Bio3 | isothermality |
| Bio4 | temperature seasonality |
| Bio5 | max temperature of warmest month |
| Bio6 | min temperature of coldest month |
| Bio7 | temperature annual range |
| Bio8 | mean temperature of wettest quarter |
| Bio9 | mean temperature of driest quarter |
| Bo10 | mean temperature of warmest quarter |
| Bio11 | mean temperature of coldest quarter |
| Bio12 | annual precipitation |
| Bio13 | precipitation of wettest month |
| Bio14 | precipitation of driest month |
| Bio15 | precipitation seasonality |
| Bio16 | precipitation of wettest quarter |
| Bio17 | precipitation of driest quarter |
| Bio18 | precipitation of warmest quarter |
| Bio19 | precipitation of coldest quarter |
