# Supplementary material for: Risk prediction of two types of potential snail habitats in Anhui Province of China: Model-based approaches
Source: PLoS Negl Trop Dis. 2020 Apr 6;14(4):e0008178. doi: 10.1371/journal.pntd.0008178 (PMC7162538; doi:10.1371/journal.pntd.0008178)
Supplement: S1 Text — (DOCX) [file pntd.0008178.s006.docx]

S1 Text Detailed introduction of models used in this study

Bioclim is the first widely applied ENM software package, and carried out by Henry Nix et al. to assess the spatial distribution pattern of crops as early as 1986 [1]. As a range-based model, Bioclim describes a series of species bioclimatic envelops through rectilinear volumes, and which are defined as “n-dimensional hyperspace”. Hutchinson raised the concept of n-dimensional hyperspace in 1957 and suggested that species can survive in the locations where all independent parameters fall within the extreme ranges determined by a set of bioclimatic envelops [2].

Domain uses a point-to-point similarity metric to make a discrimination based on the Gower distance algorithm, which uses range standardization to quantifying similarity between two sites [3]. Unlike Bioclim, Domain defines no discrete boundary for the climate envelope. The software of DIVA-GIS version 7.5(http://www.diva-gis.org/download) was used to construct two Presence-only models of Bioclim and Domain.

The basic theory of Maxent (Maximum Entropy Models) was put forward by E.T Jaynes in 1957 [4]. The model of maxent was constructed in a desktop software of maxent version 3.3.3k (Princeton University, USA, <http://biodiversityinformatics.amnh.org/open_source/maxent/>). This program only needs to input presence data along with coordinate data of each record, as well as environmental layers. The output format of “logistic” was selected and Jackknife test was chosen in this program to estimate the importance of the environmental variables. The other parameters use the default values of the program.

GARP (Genetic Algorithm for rule-set Production) is an artificial intelligence method based on genetic algorithm, which can infer correlations between known species localities and a set of environment parameters [5]. Considering that GARP is non-deterministic and every run using the same data will produce slightly different results. Desktop GARP software of version 1.1.6 (University of Kansas Center for Research, USA, <http://www.nhm.ku.edu/desktopgarp/index.html>) was used to construct the model of GARP. We set GARP to perform 100 runs with a convergence limit of 0.01 and 1000 maximum iterations. All four rule types including atomic, range, negated range and logistic regression were employed as well as the best subset feature of GARP. We then used the summation feature in the Raster Calculator of ARCGIS to make a final, cumulative predictive map.

GLM (Generalized Linear Models) are mathematical extensions of linear models, allowing for variance heterogeneity and non-linearity. For GLM model, probability distributions of response variables should obey exponential family including binomial, poisson, negative binomial, et al. The family used in our study is binomial. Since structures of most ecological data is non-normal and therefore, GLM is suited for analyzing ecological relationships.

MARS (Multiple Adaptive Regression Splines) is a tree-based modeling technique that combines classical linear regression, mathematical construction of splines, and binary recursive partitioning to produce the model. The relationships between the response and the predictions are either linear or complex non-linear. MARS can be applied to describe either linear or complex non-linear relationships between the response and the predictions through a set of “basis function”. In our study, *type* and *interaction.level args* are switched off, *penalty* is 2 and *thresh* is 0.001.

FDA (Flexible Discriminant Analysis) is a multigroup nonlinear discrimination method, which is based on non-parametric regression followed linear discriminant analysis (LDA) [6],. FDA can construct more flexible boundaries, such as quadratic and then show better classification performance.

CTA (Classification Tree Analysis) was first proposed by Breiman as a rule-based classification method [7], which generated a binary tree through “binary recursive partitioning”. CTA is not based on an assumed relationship between the response variable and explanatory variables, while just generate a binary tree to classify predictive variables through the analysis of response variable. The method of parameter used in our study is class and default rpart parms value are kept.

GBM (Generalized Boosted Model) is a flexible estimation method for fitting regression models to data, which involves an iterative process with multiple regression trees to describe the complex and non-linear relationships between the response variable and explanatory variables without over-fitting the data. The distribution used in our study is Bernoulli, the *number of tree* is 2500, the *depth of interaction* is 7 and *other parameter* set default values.

RF (Random Forest) is a combination of many classification trees and produce predictions from all the trees. The training algorithm of RF is based on bootstrap aggregating. Each tree is trained depending on the selection of many bootstrap samples, and then was evaluated through the rest data to produce more accurate classifications. The unknown class of an observation will be calculated by majority vote of the out-of bag predictions for that observation. In our study, number of trees to grow is 500, minimum size of terminal nodes is 5 and trees are grown to the maximum possibility. Number of variables randomly sampled as candidates at each split is sqrt(p) where p is number of variables in predictors.

 SVM (Support Vector Machine) is a group of supervised learning methods that can be applied to classification or regression. Support vector machines represent an extension to nonlinear models of the generalized portrait algorithm developed by [Vladimir Vapnik](http://www.clrc.rhul.ac.uk/people/vlad/index.shtml). The SVM algorithm is based on the statistical learning theory and the Vapnik-Chervonenkis (VC) dimension

Artificial neural networks (ANN) are computing systems that are inspired by, but not identical to, [biological neural networks](https://en.wikipedia.org/wiki/Biological_neural_network) that constitute animal [brains](https://en.wikipedia.org/wiki/Brain). Such systems "learn" to perform tasks by considering examples, generally without being programmed with task-specific rules. The number of units and parameter for weight decay used in our study is optimized by cross validation based on model AUC (tested decay will be the following c(0.001, 0.01, 0.05, 0.1) ). The *initial random weights* on [-0.1, 0.1] and the *maximum number of iterations* was 200 in our study.

**Reference**

1. Booth T H, Nix H A, Busby J R, et al. bioclim : the first species distribution modelling package, its early applications and relevance to most current MaxEnt studies[J]. Diversity and Distributions, 2014,20(1):1-9.

2. Elith J, Graham C H, Anderson R P, et al. Novel methods improve prediction of species' distributions from occurrence data[J]. ECOGRAPHY, 2006,29(2):129-151.

3. Carpenter G, Gillison A N, Winter J. DOMAIN: a flexible modelling procedure for mapping potential distributions of plants and animals[J]. Biodiversity and Conservation, 1993,2(6):667-680.

4. Jaynes E T. Information Theory and Statistical Mechanics[J]. Physical Review, 1957,106(4):620-630.

5. Holland J H. Genetic Algorithms[J]. Scientific American, 1992,267(1):66-72.

6. Hastie T, Tibshirani R, Buja A. Flexible Discriminant-Analysis by Optimal Scoring[J]. Journal of The American Statistical Association, 1994,89(428):1255-1270.

7. De'Ath G, Fabricius K E. Classification and regression trees: A powerful yet simple technique for ecological data analysis[J]. ECOLOGY, 2000,81(11):3178-3192.
