# Supplementary material for: Risk prediction of two types of potential snail habitats in Anhui Province of China: Model-based approaches
Source: PLoS Negl Trop Dis. 2020 Apr 6;14(4):e0008178. doi: 10.1371/journal.pntd.0008178 (PMC7162538; doi:10.1371/journal.pntd.0008178)
Supplement: S2 Text — (DOCX) [file pntd.0008178.s007.docx]

S2 Text The detail of the Investigation of Oncomelania hupensis snails in Anhui Province

In 2016, a snail survey was conducted in Anhui Province through systematic sampling and environmental sampling, covering all known snail habitats or historically recorded snail habitats and suspected environments of snail breeding [1]. In this survey, 22848 environments were surveyed from 365 townships and 2358 villages, including 3302 (14.5%) marshland and lake regions and 19546 (85.5%) hilly and mountainous regions. The total area of the surveyed environments was 1.625 billion m^2^. There were in all 1316064 frames of snails that were surveyed in the field and 170145 frames with living snails and 515867 living snails were found. The density of living snails was 0.392 snails/0.1 m^2^, and the mean rate of having living snails in the investigated frames was 12.93%, none of them were schistosome-infected snails. In the survey, there were 4830 (21.1%) environments with alive snail habitats covering an area of 0.265 billion m^2^ in 38 counties of 7 cities, including the newly emerged area of 1287.65 hm^2^ and reemerged area of 1375.32 hm^2^. In addition, there were 17927 historical recorded snail habitats and 91 suspected environments in whole province. Among the 4830 environments with alive snail habitats, there were 1080 marshland and lake regions (22.4%) and 3750 (77.6%) hilly and mountainous regions. Our data used in this study is sampled randomly from the environments with snail habitats through ArcGIS10.

**Reference**

1. Gao FH, Zhang SQ, etc. Investigation of Oncomelania hupensis snails in Anhui Province in 2016 [J]. Chinese Journal of Schistosomiasis Control, 2018, 30(5):493-499.
